# Deletions in *cox2* mRNA Result in Loss of Splicing and RNA Editing and Gain of Novel RNA Editing Sites 

**DOI:** 10.1371/journal.pone.0082067

**Published:** 2013-12-04

**Authors:** Stefanie Grüttner, Christina Hopf, Abhishek Kumar, Frank Kempken

**Affiliations:** Abteilung für Botanische Genetik und Molekularbiologie, Botanisches Institut und Botanischer Garten, Christian-Albrechts-Universität zu Kiel, Kiel, Germany; NIGMS, NIH, United States of America

## Abstract

As previously demonstrated, the maize *cox2* RNA is fully edited in cauliflower mitochondria. Use of constructs with a deleted *cox2* intron, however, led to a loss of RNA editing at almost all editing sites, with only a few sites still partially edited. Likewise, one deletion in exon 1 and three in exon 2 abolish RNA editing at all *cox2* sites analyzed. Furthermore, intron splicing is abolished using these deletions. Mutation of a cytosine residue, which is normally edited and localized directly adjacent to the intron, to thymidine did not result in restoration of splicing, indicating that the loss of splicing was not due to loss of RNA editing. One deletion in exon 2 did not lead to loss of splicing. Instead, most editing sites were found to be edited, only three were not edited. Unexpectedly, we observed additional RNA editing events at new sites. Thus it appears that deletions in the *cox2* RNA sequence can have a strong effect on RNA processing, leading to loss of splicing, loss of editing at all sites, or even to a gain of new editing sites. As these effects are not limited to the vicinity of the respective deletions, but appear to be widespread or even affect all editing sites, they may not be explained by the loss of PPR binding sites. Instead, it appears that several parts of the *cox2* transcript are required for proper RNA processing. This indicates the roles of the RNA sequence and structural elements in the recognition of the editing sites.

## Introduction

Mitochondrial RNA editing in higher plants has long been described [1–3], and is discussed in recent reviews [4–8]. In short, RNA editing is characterized mostly by C-to-U changes, with rare U-to-C editing, targeting hundreds of RNA editing sites in plant mitochondrial transcripts, and to a lesser degree also in higher plant plastids. Sequences close to the editing sites are required for RNA editing. Employing a wheat *in organello* approach, a region of 16 nucleotides upstream and six nucleotides downstream was shown to be required for recognition of two *cox2* editing sites [9,10]. Using an *in vitro* RNA editing system isolated from pea mitochondria a 20-nucleotide region upstream of the first *atp9* editing site was shown to be essential. However, for efficient editing, an upstream sequence of 40 nucleotides was required [11]. A cauliflower *in vitro* assay not only confirmed this result, but identified one nucleotide downstream of the *atp9* editing site as essential [12]. 

In recent years nuclear encoded mitochondrial or plastid proteins have been identified, which are required for RNA editing of specific sites. Almost all belong to the pentatricopeptide repeat protein family, which has 450 members in *Arabidopsis thaliana*. These proteins are characterized by 4-20 repeats of 34-36 amino acids each [13]. At current 18 mitochondrial and 15 plastid PPR proteins involved in an RNA editing site determination have been identified and reviewed [4–8]. All of them belonging to the PLS subfamily of PPR proteins which is characterized by triplets of 35, 36 and 31 amino acid motifs. Most of these proteins also possess C-terminal domains, which are named E, E+, or DYW [14]. All PPRs implicated in editing carry at least an E domain, several have an additional DYW domain, which contains amino acid motifs characteristic for zinc containing cytidine deaminases [15]. Indeed, the editing factor MEF1 is only functional with its DYW domain [16]. However, the DYW domain appears not to be essential for all mitochondrial RNA editing events, as the MEF11 protein is functional without it [4]. While some of these PPRs recognize single editing sites, other target several editing sites. For example, the DYW protein MEF11 targets editing sites in the *ccb203*, *cox3*, and *nad4* transcripts [17–19]. Recently, an RNA editing factor interacting protein 1 (RIP1) was identified, which is localized in plastids and mitochondria. RIP1 mutants affect all known *A. thaliana* plastid editing sites, and hundreds of mitochondrial ones [20]. Likewise, a multiple organellar RNA editing factor (MORF) family was identified, members of which are required for plastid and mitochondrial editing. The loss of a MORF protein abolishes or lowers editing at multiple sites [21]. Neither RIP1 nor MORF belong to the PPR family, but appear to be important components of the RNA editing machinery. 

Current data suggest that the target RNA motif of PPR editing factors is rather little defined, resulting in a network of PPR trans-factors which bind to RNA sequences at a low level of specificity [4]. Such a network of factors may explain how some 140 E-domain PPRs are sufficient for editing of more than 400 editing sites [4], even if some editing factors target the same sites. Indeed, very recently it was reported that two E domain PPR, MEF8 and MEF8S target the same two editing sites [22]. Hence, it remains elusive how editing site specificity is obtained.

We previously had established and described *in organello* systems for mono- and dicotyledonous plants. These systems were successfully employed to analyze mitochondrial mRNA editing and the ability of plant mitochondria to recognize and correctly process RNAs from different taxa [23–27]. In addition, we were able to demonstrate complete *in organello* editing and splicing of *in vitro* transcribed *cox2* mRNA [28].

Previous work did indicate a potential role of the secondary or tertiary structure of the RNA on editing site recognition [24]. Hence, we expanded our *in organello* system to analyze the effect of different sized deletions in the *cox2* coding sequence on RNA splicing and editing. We show that deletions in the RNA sequence can have a strong effect on RNA processing, leading to loss of splicing, loss of editing at all sites, or even to a gain of new editing sites. 

## Results

A growing number of PPR proteins have been implicated in editing site recognition. However, their target motifs appear to be little defined [4]. In previous work, we found limited evidence for involvement of the secondary or tertiary structure of an mRNA in the editing process by comparison of editing site recognition of *cox2* mRNA from mono- and dicot origin [24]. One way to further test this possibility is to introduce deletions in the mRNA sequences. If RNA editing is solely due to site-specific binding of PPR proteins or PPR protein networks such deletions should have an effect at editing sites directly adjacent to the deletion at best. Any observed long-range effect provides direct evidence for an involvement of the secondary or tertiary structure of the mRNA on editing site recognition.

**Figure 1:**
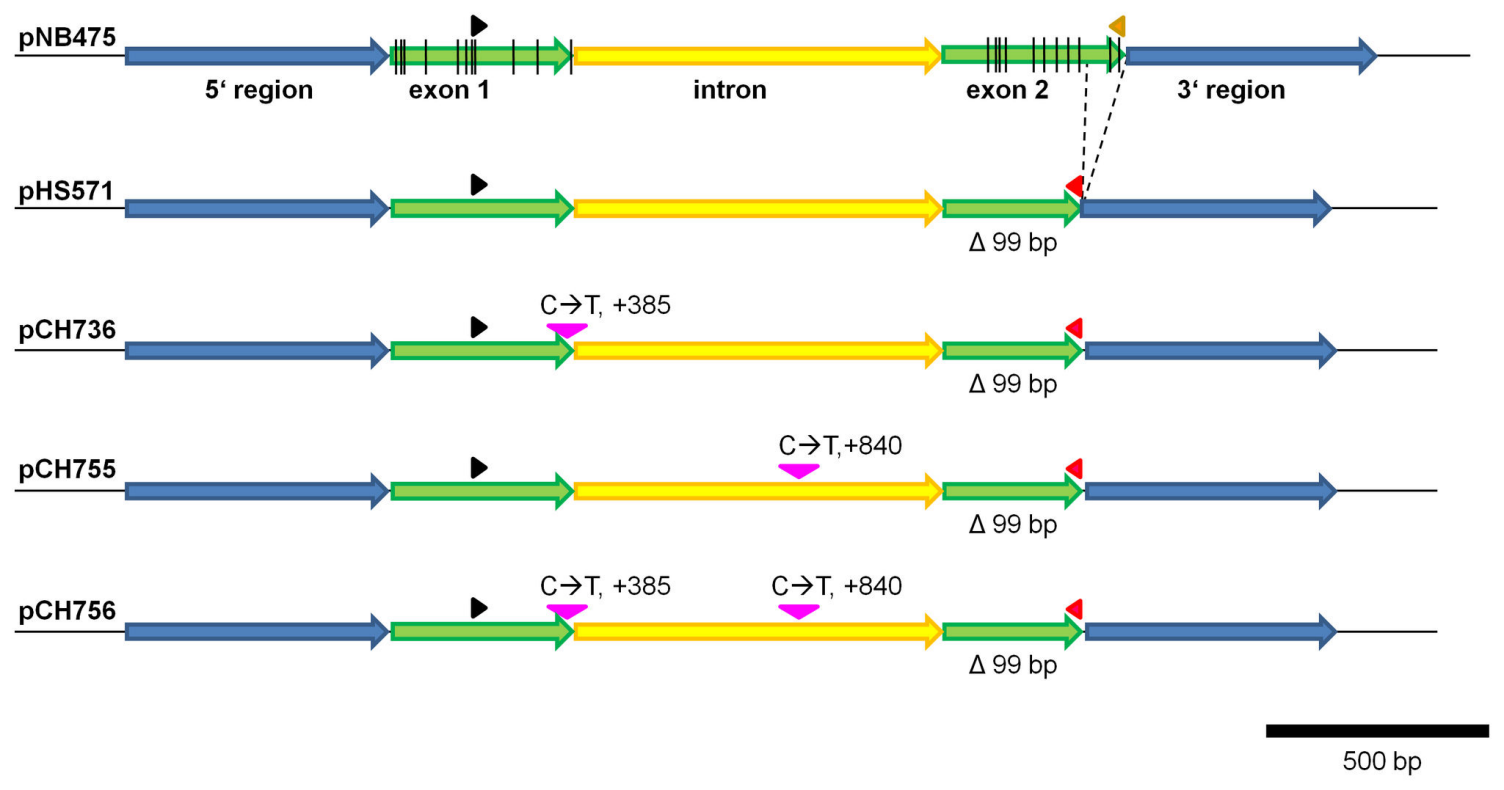
Vectors pHS571 and mutated derivatives. Both *cox2* exons (green) and the intron (yellow) were amplified from *Zea mays* mitochondrial DNA, while the 5´ and 3´ regions (blue) originate from *A. thaliana*. Vertical lines in exons indicate RNA editing sites. Black arrowhead: oligonucleotide NB852, orange arrowhead: oligonucleotide FK789; red arrowhead: oligonucleotide IH977, which were used for specific RT-PCRs; pink arrowhead: mutated positions in the constructs pCH736, pCH755, and pCH756; vector names are indicated at the left hand side. Deletions are indicated in size (bp = base pairs) and using dotted lines.

**Figure 2:**
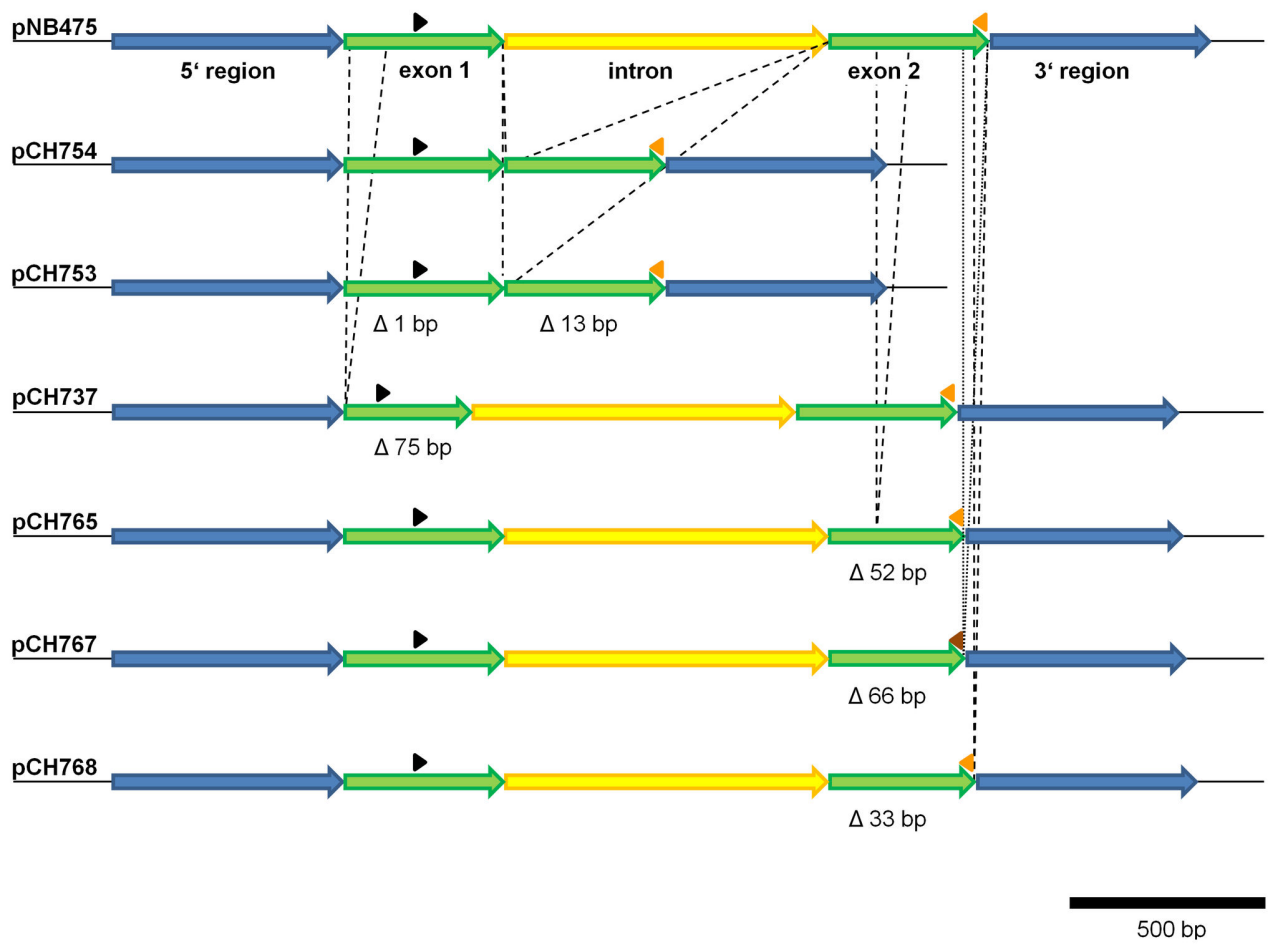
RT-PCR and sequence analysis using vectors pNB475 and pHS571. (A) RT-PCR of transcripts from pNB475 showing the correct size of spliced *cox2* mRNA.Expected size for mRNA: sp – 567 bp; usp – 1362 bp. (B) The size of the RT-PCR amplicon from transcripts of pHS571 indicates lack of splicing. Expected size for mRNA: sp – 535 bp; usp – 1328 bp. (C) Electropherogramms from sequence analysis of amplicons from (A) showing clear evidence for full RNA editing (green boxes). (D) Electropherogramms from sequence analysis of amplicons from (B) giving no trace for RNA editing (red boxes). In each case editing site numbering is indicated (see Figure 7 for reference). sp = spliced transcript; usp = unspliced transcript (for all figures).

**Figure 3:**
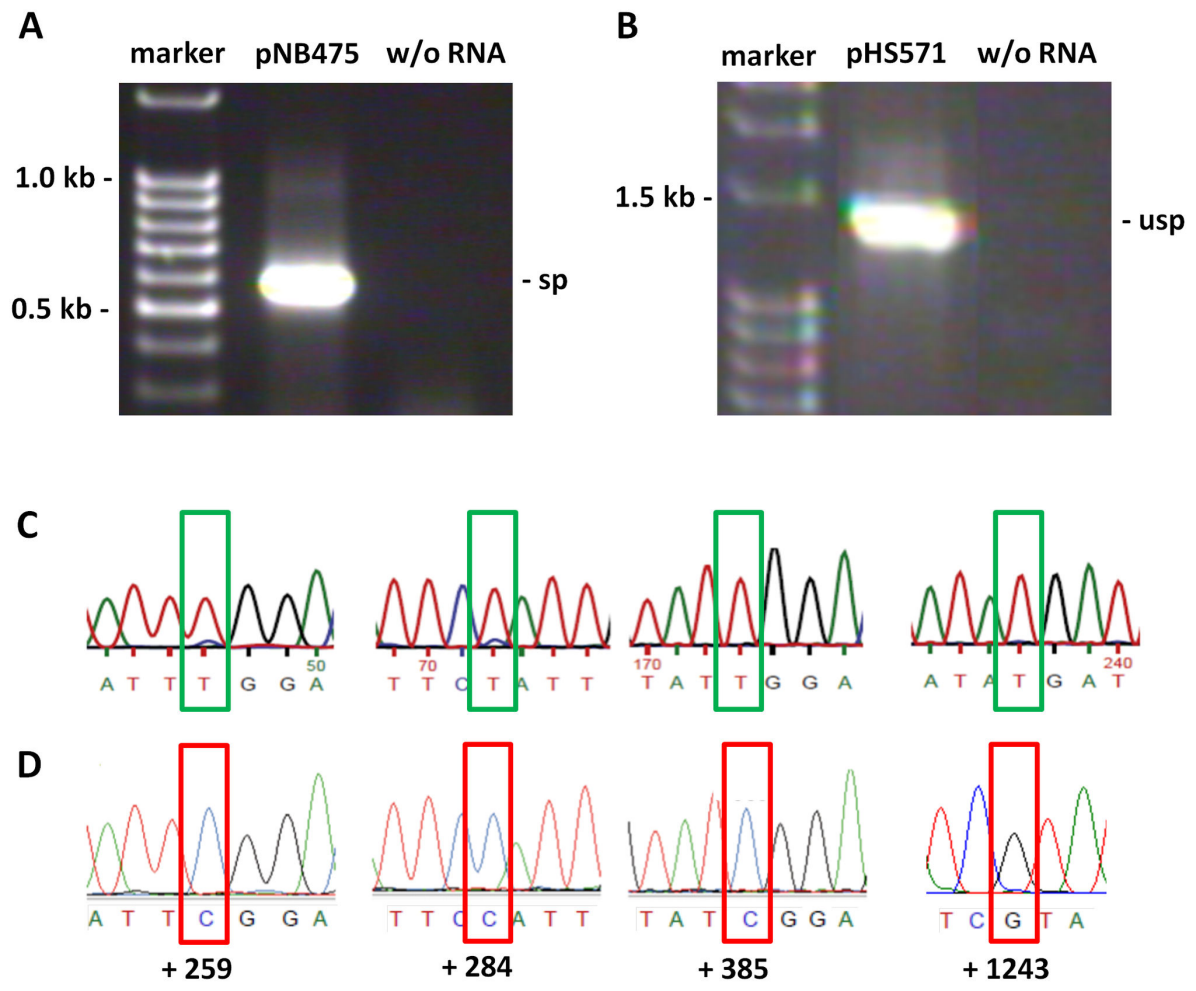
Analysis of mutated derivatives of pHS571. (**A**) RT-PCR of mRNAs from vectors pCH736, pCH755 and pCH756 (see Figure 1 and Table S2 in File S1). Expected sizes for mRNA: sp – 534 bp; usp – 1328 bp (for all constructs) (**B**) Electropherogramms from pCH736 transcripts. Editing site at position +385 was mutated from C-to-T on the DNA level. (**C**) Electropherogramms from pCH755 transcripts. (**D**) Electropherogramms from pCH756 transcripts. In each case editing site numbering is indicated. Red box: no editing.

To this end a vector, pHS571, carrying a 99 bp deletion carrying two editing sites in exon 2 (see Figure 1), was constructed and introduced into isolated mitochondria from cauliflower. Upon *in organello* incubation, mRNA was isolated and RT-PCR was performed. While the control vector pNB475 gave rise to an mRNA which was spliced (Figure 2A), pHS571 (exon2∆99 bp) mRNA apparently was not spliced (Figure 2B). This was confirmed by sequence analysis. More importantly, while pNB475 mRNA was fully edited at all sites analyzed (Figure 2C), pHS571 (exon2∆99 bp) mRNA was not edited at any site investigated (Figure 2D). The fact that pHS571 (exon2∆99 bp) mRNA was neither edited nor spliced may be explained by editing being a prerequisite of splicing. In fact, editing site 11 at position +385 is localized in the intron binding site 1 adjacent to the intron and thus editing may be required in order to allow for splicing. In addition, we sequenced non-spliced mRNA (Figure S1) to identify any editing site, which may be present in the intron sequence itself. Indeed, we found a partial editing event at position +840 in one of the six helical domains (domain II, [29]) of the intron. Partial RNA editing indicates an editing site is not edited in all mRNA molecules of the amplified pool. Hence, we used site-specific mutation to generate from pHS571 (exon2∆99 bp) plasmid pCH736 with a C-to-T mutation at position +385, plasmid pCH755 with a C-to-T mutation at position +840, and plasmid pCH756 (shown in Figure 1) carrying both mutations to analyze whether lack of editing at these sites was causative for the observed loss of splicing. As shown in Figure 3A, all constructs were transcribed, but neither was spliced nor edited (Figure 3B-D). It appears that loss of splicing is not due to loss of editing at either position +385 or +840. 

**Figure 4:**
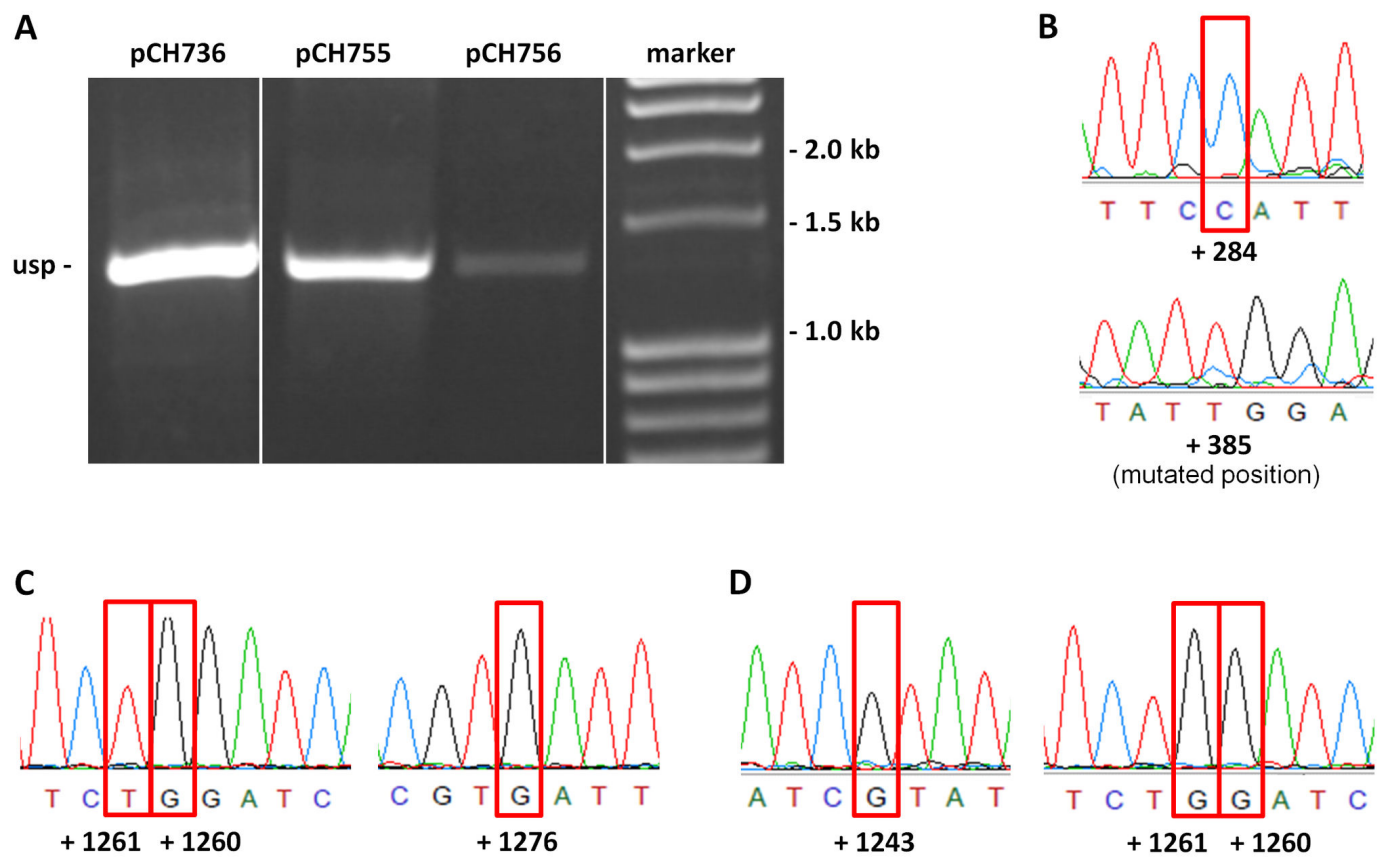
Vectors with different deletions in the *cox2* sequence. Set of eight vectors carrying different deletions, either in exon 1, exon 2 or regarding the intron. Color codes are as in Figure 1. Red arrowhead: oligonucleotide FK789, which binds to the 5´ region was used for PCR. Brown arrowhead: oligonucleotide CH2385, which binds to the 5’ region was used for PCR of pCH767 only. Deletions are indicated in size (bp = base pairs) and using dotted lines.

We then generated a larger collection of deletions shown in Figure 4. Care was taken to generate different sized deletions in each exon without removing many editing sites and not causing a frame shift mutation (pCH737 (exon1∆75 bp), pCH765 (exon2∆52 bp), pCH767 (exon2∆66 bp), and pCH768 (exon2∆33 bp)). In addition, we removed the intron from the gene (pCH754). Plasmid pCH753 exhibits the loss of the intron, and due to an unexpected PCR error the loss of one base pair from exon 1, and 13 base pairs from exon 2, thus resulting in a frame shift mutation. 

**Figure 5:**
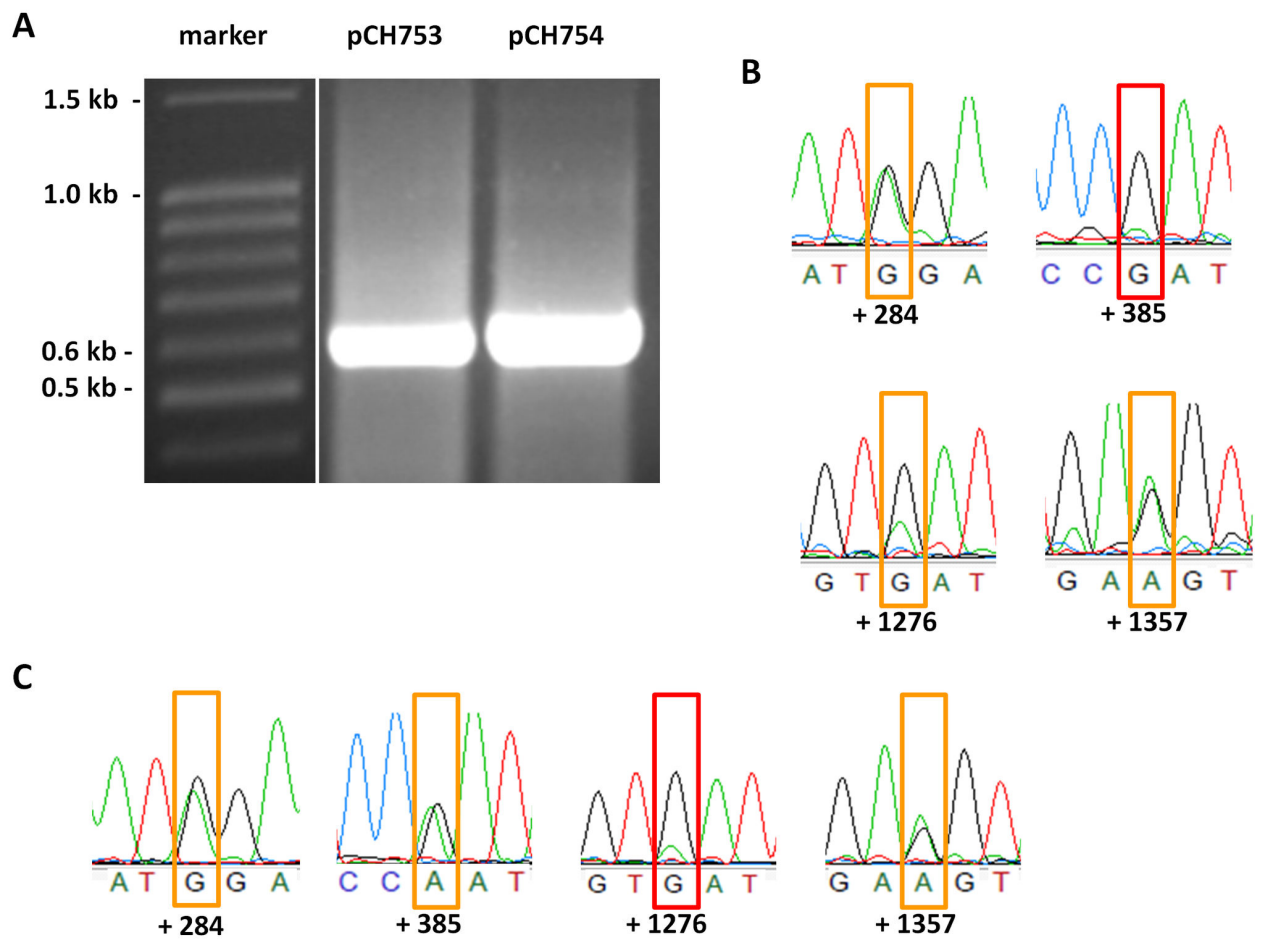
Vectors without an intron or even with a frame shift yield stable RNA. (**A**) RT-PCR of mRNAs from vectors pCH753 and pCH754, the latter carrying a frame shift mutation. (**B**) Electropherogramms from RT-PCR amplicons obtained from pCH753 mRNA. Editing site at position +284 shows partial editing and at position +385 no editing at all. (**C**) Electropherogramms from RT-PCR amplicons obtained from pCH754 mRNA. Editing site at position +1276 shows a trace of editing and at position +1357 partial editing. Red boxes: no editing; orange boxes: partial editing.

Results for pCH753 (intronless with frameshift) and pCH754 (intronless) are given in Figure 5. Both plasmids gave rise to RT-PCR products indicating transcription (Figure 5A). Editing at most sites is lost in transcripts from both plasmids (Figure 5B, C), however, editing sites at position +284, +1276, +1357 and +1381 remain partially edited in transcripts from pCH753 (intronless with frameshift) as well as editing sites +284, +385, +1276, +1357, +1381 and +1414 in transcripts from pCH754 (intronless) (see Figure 5B, C). 

**Figure 6:**
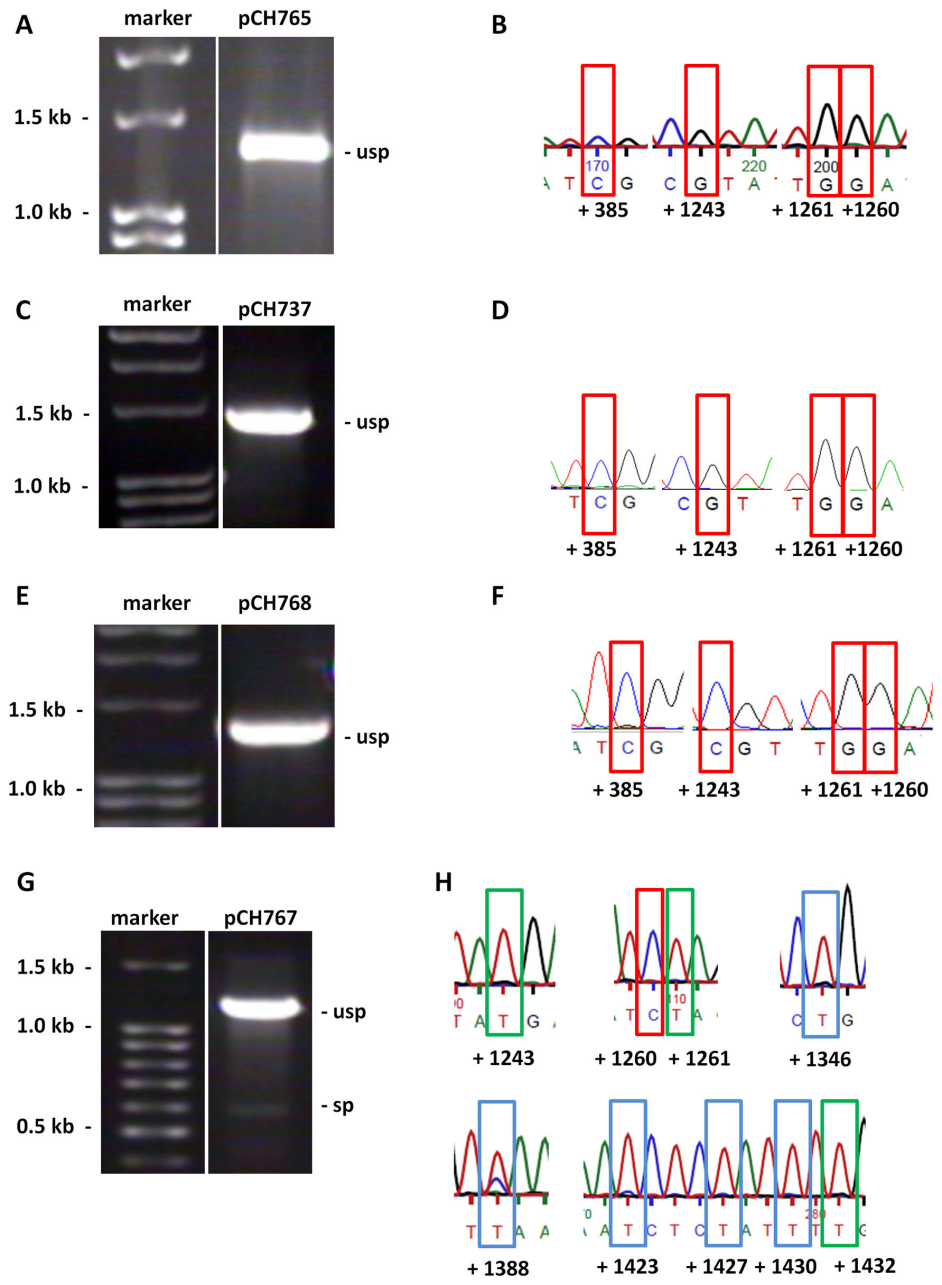
Analysis of mRNAs from vectors with deletions in exon 1 or 2. (**A**, **B**) RT-PCR of mRNA from pCH765 and examples of corresponding electropherogramms. Expected size for mRNA: sp – 518 bp; usp – 1311 bp. (**C**, **D**) Same for pCH737. Expected size for mRNA: sp – 569 bp; usp – 1362 bp. (**E**, **F**) Same for pCH768. Expected size for mRNA: sp – 536 bp; usp – 1329 bp. (**G**, **H**) Same for pCH767. Expected size for mRNA: sp – 501 bp; usp – 1294 bp. Red box: no editing; green box: full editing; blue box: new editing site.

**Figure 7:**
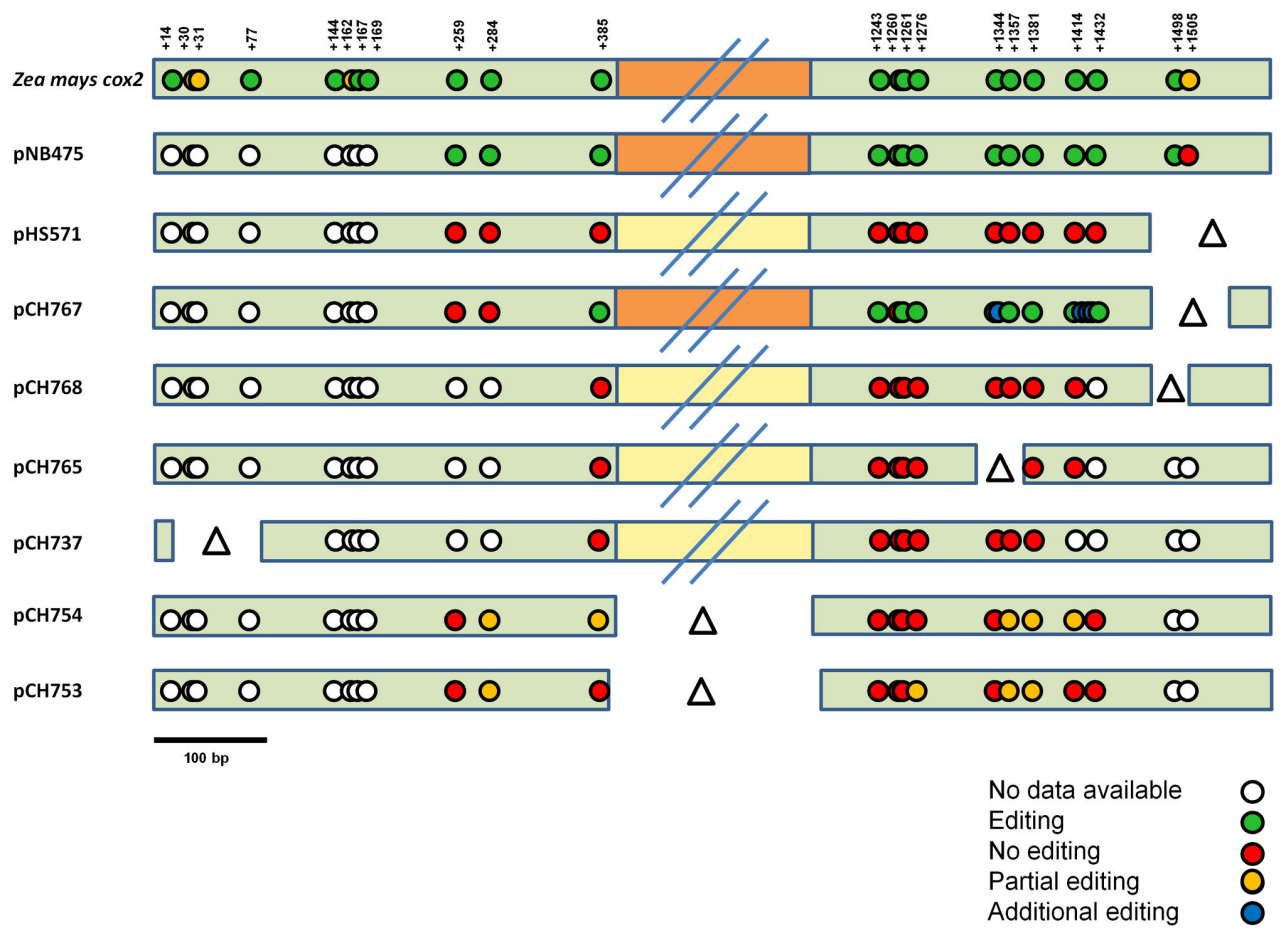
Comparison of editing status of all mRNAs analyzed. Editing status of the endogenous maize *cox2* and the different vectors used in this study. White dot – no data available; green dot – site fully edited; red dot – no editing observed; orange dot – partial editing; blue dot – new editing site; Δ – deletion; exons are shown as green bars, the intron as yellow bar, for spliced transcripts the intron is shown as orange bar; the intron is not shown to scale.

The other plasmids carrying deletions in exons and shown in Figure 4 gave different results. While transcripts from pCH737 (exon1∆75 bp), pCH765 (exon2∆52 bp) and pCH768 (exon2∆33 bp) were neither spliced nor edited (Figure 6A-F), in contrast transcripts from pCH767 (exon2∆66 bp) were spliced and edited (see Figure 6G, I). However, for these constructs we did not observe full splicing. The unspliced amplicon did not exhibit any evidence for RNA editing (Figure S2). Spliced amplicons were further tested. Surprisingly, for plasmid pCH767, carrying a 66 bp deletion in exon 2, five new editing sites occurred at positions +1346, +1388, +1423, +1427, and +1430 (see Figure 6H and Figure 7). Site +1388 was edited only in some experiments, but not in all as the other sites. We did compare the 5´and 3´sequences adjacent to these new editing sites, however no obvious similarities could be detected. (Figure S3).

The data obtained for pHS571 (exon2∆99 bp) (Figure 7) hint to the presence of a sequence motif within nucleotides +1477 to +1577 essential for RNA editing. Indeed using pCH767 (exon2∆66 bp), which lacks the last 66 bp of exon 2 is the only one still showing RNA editing activity (Figure 7). However, there are additional sites edited using this construct, which never have been observed before. However, using pCH768 (exon2∆33 bp) (Figure 7) no editing and splicing occurred although this construct carries an even larger section of the part deleted in pHS571 (exon2∆99 bp). Thus it appears unlikely that specific nucleotide sequences present in exon 2 are required for RNA editing of the *cox2* mRNA. 

Hence, CLC Genomics Workbench was used to obtain secondary structure data for *cox2* mRNA and all deletion constructs used. These data are shown in Figure S4, which provides a possible explanation how sequence and structural changes in *cox2* mRNA affects RNA editing. pNB475 and pHS571 (exon2∆99 bp) differ in 3’ deletion of one bulge, two hairpin and two interior loops and three stems (Table S3A in File S1).   Furthermore, differences in RNA secondary structural elements are illustrated on global level as shown in Figure S2 and shown in Table S3B-E in File S1.   This corroborates that a combination of these structural elements served as switch on/off for the RNA editing process in RNA from different mutated plasmids.

## Discussion

Several studies employing either *in vitro* or *in organello* RNA editing systems have provided important evidence on the process of mitochondrial and plastid RNA editing [23,26–28]. Even more relevant, several pentatricopeptide repeat proteins of the PLS subfamily are essential for editing of specific RNA editing sites [4–8]. Furthermore, the MORF family provides additional components of the RNA editing machinery [21]. Some PPR editing factors recognize different editing sites with divergent surrounding nucleotide sequences thus raising the question of how specificity of editing site recognition is managed [17,19]. It is noteworthy that even contiguous editing sites may be recognized by two different PPR proteins [30]. Hence the existence of a network of PPR trans-factors was suggested which bind to RNA sequences at a low level of specificity [4]. This may explain the discrepancy of how 140 potential PPRs may bind to 400 editing sites. However, the data present here demonstrate that deletions in the exons or removal of the intron do not influence editing sites adjacent to the deletion. Instead, in the cauliflower *in organelle* system, they drastically reduce RNA editing at all sites of the maize *cox2* mRNA. The size of these deletions varied from 27 to 99 base pairs in either exon. All but one abolished editing and splicing completely. In contrast, some partial edited sites remained upon removal of the *cox2* intron. A similar observation was made using a wheat *in organello* system and employing potato *rps10* and wheat *cox2* mRNA, where removal of the respective introns led to loss or partial loss of RNA editing in the transcripts [31]. The study also confirmed earlier observations [32,33] that editing of certain sites in or close by an intron is a prerequisite of splicing, although in some cases editing may occur after splicing [34]. This data were interpreted as an indication of a close linkage of editing and splicing factors during RNA processing [31]. In the study current, it was not possible to reestablish RNA splicing by *in vitro* mutation of editing sites close-by or in the intron. However, it is possible that we missed additional editing sites in the intron sequence, although not previously reported from other *cox2* mRNAs. An alternative explanation is provided by changes in the RNA secondary structures of pCH737 (exon1∆75 bp), pCH765 (exon2∆52 bp), and pCH768 (exon2∆33 bp) (see Figure S2), which have additional bulges and interior loops in the proximity of the intron binding sites, which may explain loss of splicing, as the proper RNA secondary structure is required for splicing [29,35].

If indeed PPR editing factors recognize editing sites either on their own or acting in a network, one might argue that deletions should not have an effect on distant editing sites. Instead only local effects on neighboring sites might be expected, e.g. due to loss of the PPR binding sites. In this study all but one deletion had a global effect on maize *cox2* mRNA editing in cauliflower, which is not readily explained by the current knowledge on mitochondrial editing factors and their interaction. The first example of loss of editing involving all editing sites of a transcript was observed in *atp6* mRNA in cytoplasmic male sterile *Sorghum bicolor* [36], although in a later study a reduction but not total loss in editing efficiency was observed only [37]. While no mechanistic explanation was presented then, later experiments employing a cauliflower *in organello* editing system and maize *cox2* mRNA and vice versa hinted to a function of the mRNA structure in the editing process [24]. Cauliflower and maize mitochondria were able to edit all sites of the opposite *cox2* mRNA even if the editing sites are absent in their own endogenous mRNA. Most intriguingly two monocot-specific editing sites, which are pre-edited on the DNA level in dicots, are properly edited in cauliflower mitochondria [24]. Additional evidence was obtained from the analysis of RNA editing of *atp6* mRNA from *Sorghum bicolor* in maize mitochondria. RNA editing depended on the presence of part of the 5´ UTR and the open reading frame from maize in a chimeric transcript [26]. These data cumulatively let to speculate about a self-guided RNA editing model consisting of PPR editing factors recognizing secondary or tertiary RNA structural motifs [27]. This model is supported by the data presented here, as all but one deletion has a global effect on *cox2* mRNA editing, which may be explained best by changes in the RNA secondary structure. Unfortunately, RNA secondary or tertiary structure predictions by means of bioinformatics are still rather crude and results obtained are hard to confirm or evaluate. The structural predictions presented in this study provide hints that both sequence and structural elements are essential for RNA editing. Similar results are reported for ADAR2 based RNA editing that converts adenosine to inosine (A I) in mammals [38]. However, it requires separate investigations with further development in computational prediction for RNA structural predictions. 

## Experimental Procedures

### Oligonucleotides

All oligonucleotides are shown in Table S1 in File S1. Oligonucleotides CH2279, CH2280, CH2358 and CH2359 (Eurofins MWG Operon, Ebersberg) were used for *in vitro* mutagenesis. Oligonucleotides CH2294, CH2322, CH2328, CH2337, CH2338, CH2355, CH2356, CH2381, CH2382, CH2383, CH2385, CH2386, CH2387 were employed in order to introduce deletions into the different vectors. Oligonucleotides FK864 and FK865 were used for construction of vector pHS571. Oligonucleotides FK 770, FK789, NB852, IH977, CH2319 through CH2322, and CH2385 were used for specific amplification of RNA transcribed from the introduced vectors. 

### Vectors

All plasmids are derivatives of plasmid pNB475 [24], which contains exon 1, the intron, and exon 2 of the maize *cox2* gene under control of the *Arabidopsis thaliana cox2* promoter and 3´ region. Plasmids pHS571, pCH737, pCH753, pCH754 and pCH765, pCH767, and pCH768 are derived from pNB475 by PCR based deletion using the respective oligonucleotides listed in the table. Plasmids pCH736, pCH755 and pCH756 were generated by *in vitro* mutagenesis of pHS571. All vectors are listed in Table S2 in File S1 and shown in Figure 1 and Figure 4.

### Plant material

Cauliflower heads were obtained from local grocery stores or grown in greenhouses in the Botanical Garden of the Christian-Albrechts-University (CAU) at Kiel.

### Mitochondrial electroporation and *in organello* incubation

All mitochondrial procedures were performed as described [23,39], however, no DNAse treatment was performed during *in organello* incubation and 1200 µg mitochondrial protein was used. *In organello* incubation of mitochondria was carried out for three hours, as described previously [23,39].

### Nucleic acid isolation and gel electrophoresis

Mitochondrial RNA was isolated as described [23]. Quantification of RNA was done using a Nanodrop ND-1000 (Peqlab, Thermo Fisher Scientific) provided by the Center of Molecular Biology of CAU (Kiel). Bacterial plasmid DNA was isolated using either a NucleoBond Reagent Set (Macherey-Nagel, Düren) or the Plasmid Midi Kit (QIAGEN, Hilden).

### PCR, RT-PCR and sequence analysis

These procedures were performed as described previously [24] using an One-step RT-PCR kit from Qiagen (Hilden).

### PCR-based site-directed mutagenesis

The methodology is based on the QuikChange Site-Directed Mutagenesis Kit and the ExSite PCR-Based Site-Directed Mutagenesis Kit (Stratagene). For specific *in vitro* introduction of point mutations and deletions into plasmid DNA recombinant PCR-based site-directed mutagenesis was applied. In the case of *in vitro* mutagenesis, two complementary oligonucleotides containing the desired mutation served as primers for the amplification of the whole plasmid with proofreading polymerase Pwo (Peqlab, Erlangen). Deletions were achieved using two phosphorylated inverse primers directly flanking the desired deletion for the amplification of the remaining plasmid. As template for the PCR reaction 25-50 ng plasmid DNA was employed. The PCR product was digested with Dpn*I* (New England Biolabs, Ipswich), which selectively hydrolyzed the methylated original template DNA. PCR product DNA carrying a point mutation was transformed directly into *E. coli* and recombined *in vivo* while the linear plasmid DNA of the *in vitro* deletion had to be ligated first via its phosphorylated blunt ends.

### RNA secondary structure prediction

CLC Genomics Workbench (www.clcbio.com) was used for RNA structural analysis which uses a dynamic programming algorithm for free energy minimization which includes free energy increments for coaxial stacking of stems when they are either adjacent or separated by a single mismatch [40]. The thermodynamic energy parameters were taken from the Mfold version 3 [41].

### Sequence logo construction

Sequence logo of sequence flanking RNA editing sites was created using WebLogo 3.3 [42].

### Standard procedures

All other standard molecular biology techniques were performed according to published standard procedures [43].

## Supporting Information

Figure S1 **Intron mRNA sequencing.** (A) Oligonucleotide pairs CH2319/CH2320, and CH2321/CH2322 were used to amplify unspliced mRNA. Cox2 exons are shown in green and the intron in yellow color. (**B**) Editing site 11 (in exon 1) exhibits partial editing, which is expected in unspliced mRNA. (**C**) Evidence for partial editing of one position (+840) in the intron (reverse sequencing).
(TIF)

Figure S2 **Sequencing of unspliced amplicon from Figure 6G.** No evidence for RNA editing is observed.
(TIF)

Figure S3 **Sequences adjacent to new editing sites observed for spliced mRNA from pCH767.** Sequence logo of sequence flanking RNA editing sites was created using WebLogo 3.3 [42]. No obvious sequence motifs are present.
(TIF)

Figure S4 **Models for RNA secondary structure.** (**A**-**H**) Editing status of RNA from different plasmids and comparison with RNA secondary predictions.
(PDF)

File S1 **This file contains all supplementary tables.**
(DOCX)
