# Supplementary figures and images for: Deletions in cox2 mRNA Result in Loss of Splicing and RNA Editing and Gain of Novel RNA Editing Sites
Source: PLoS One. 2013 Dec 4;8(12):e82067. doi: 10.1371/journal.pone.0082067 (PMC3852756; doi:10.1371/journal.pone.0082067)

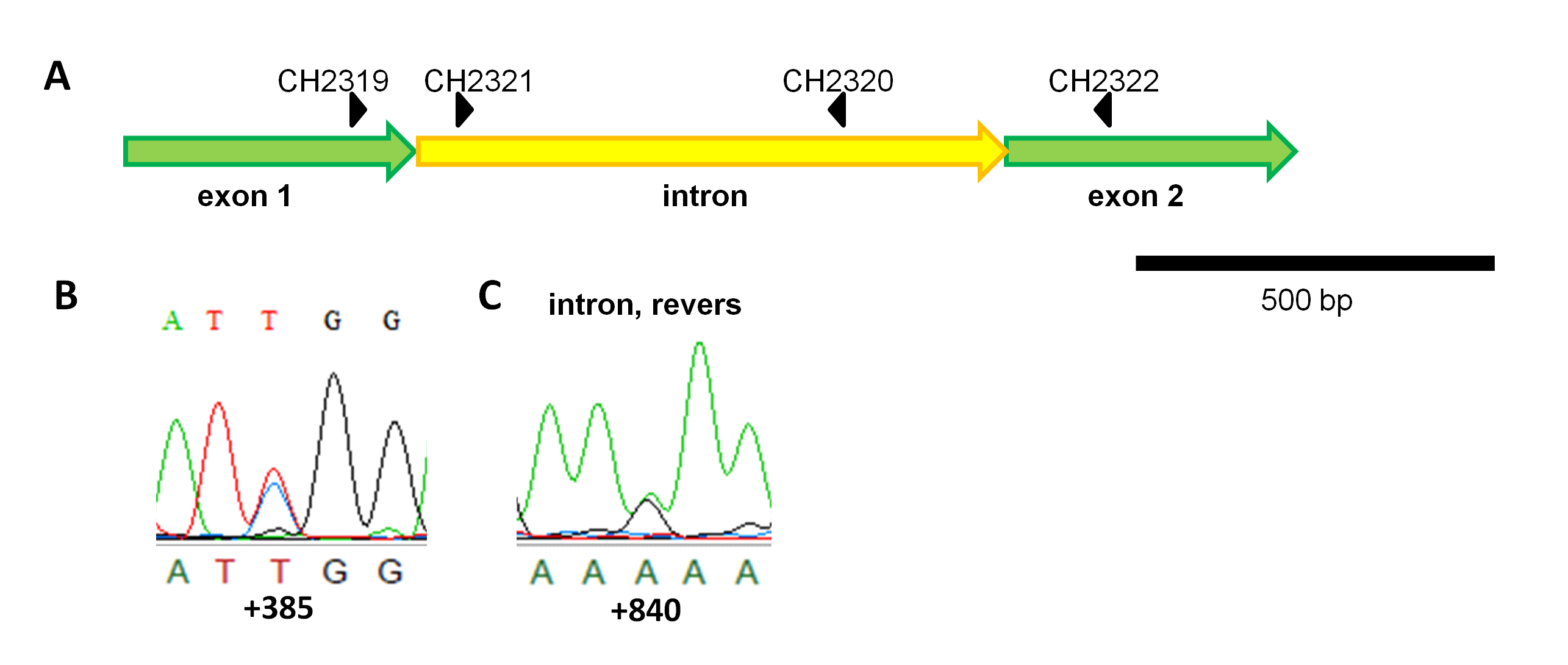

Supplement: Figure S1 — Intron mRNA sequencing. (A) Oligonucleotide pairs CH2319/CH2320, and CH2321/CH2322 were used to amplify unspliced mRNA. Cox2 exons are shown in green and the intron in yellow color. (B) Editing site 11 (in exon 1) exhibits partial editing, which is expected in unspliced mRNA. (C) Evidence for partial editing of one position (+840) in the intron (reverse sequencing). (TIF) [file pone.0082067.s001.tif]

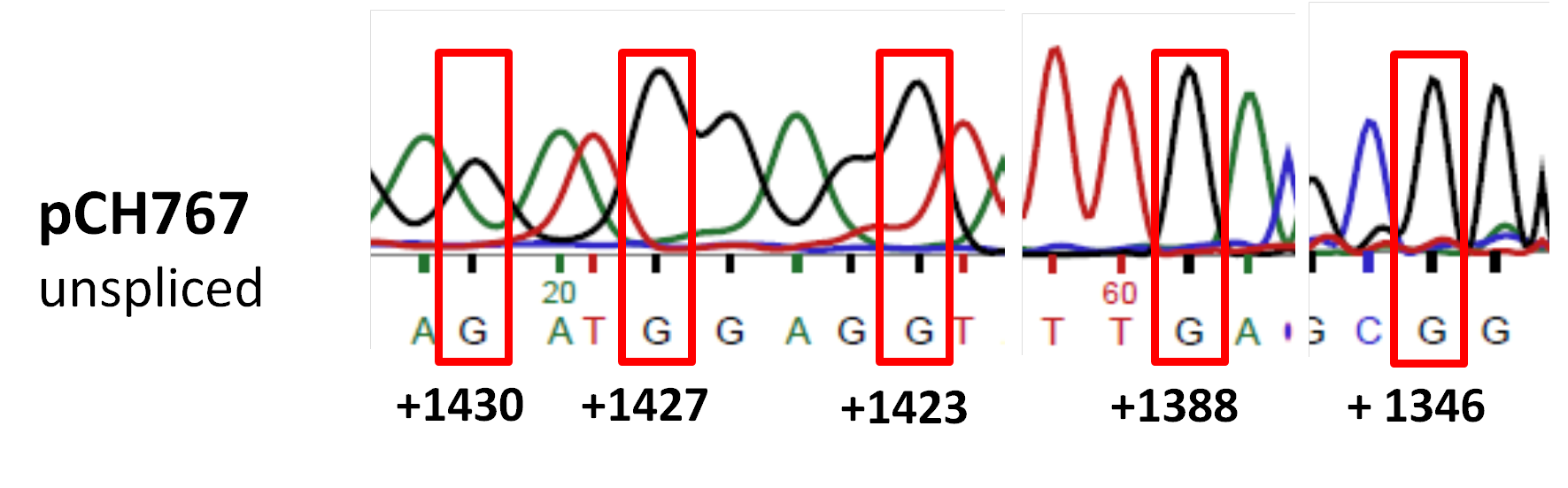

Supplement: Figure S2 — Sequencing of unspliced amplicon from Figure 6G. No evidence for RNA editing is observed. (TIF) [file pone.0082067.s002.tif]

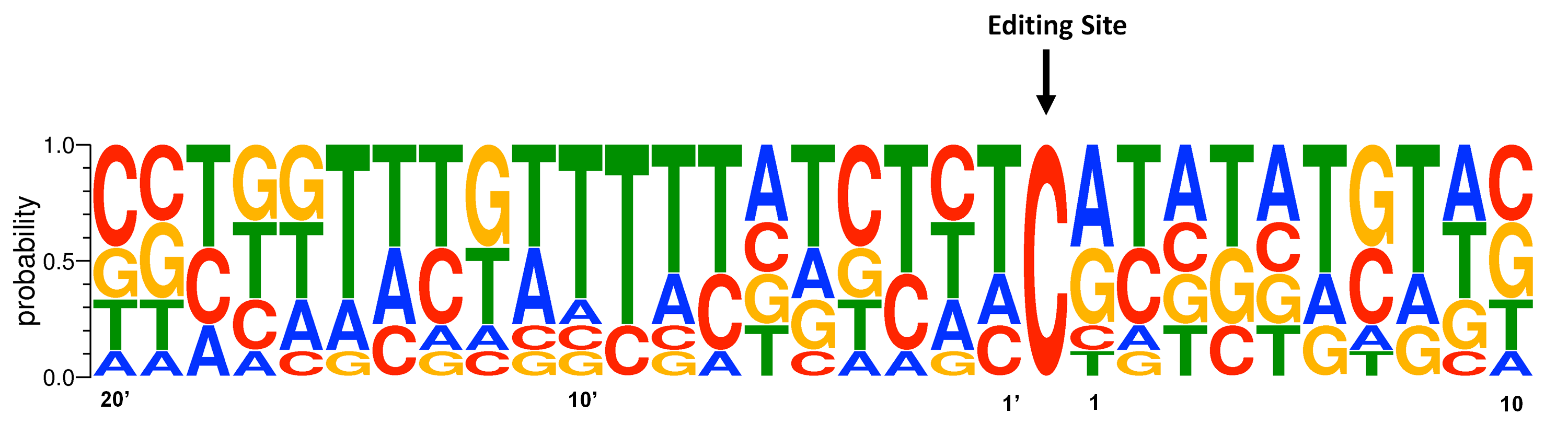

Supplement: Figure S3 — Sequences adjacent to new editing sites observed for spliced mRNA from pCH767. Sequence logo of sequence flanking RNA editing sites was created using WebLogo 3.3 [42]. No obvious sequence motifs are present. (TIF) [file pone.0082067.s003.tif]

**A**

pNB475

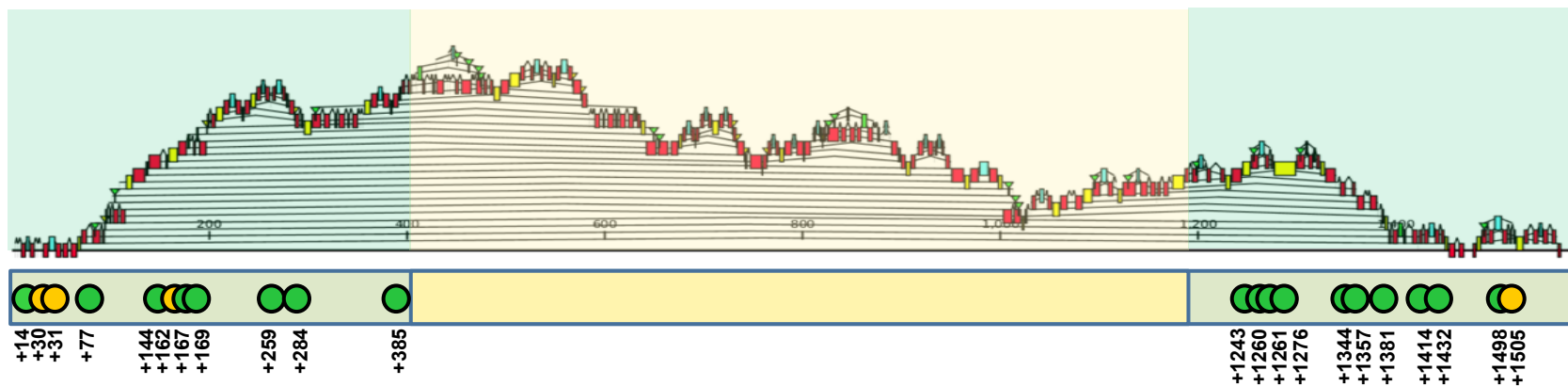**B**

pHS571

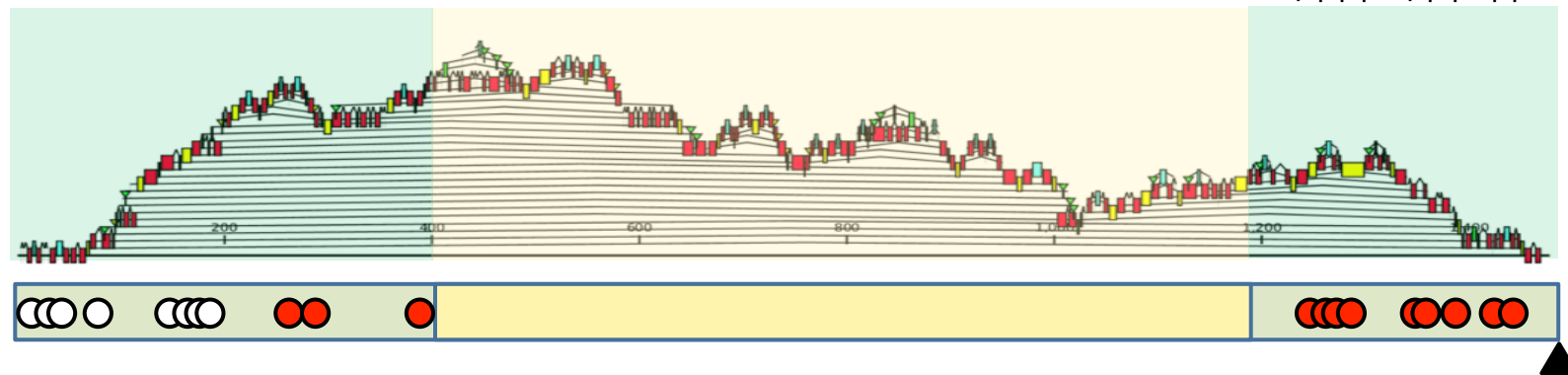**C**

pCH767

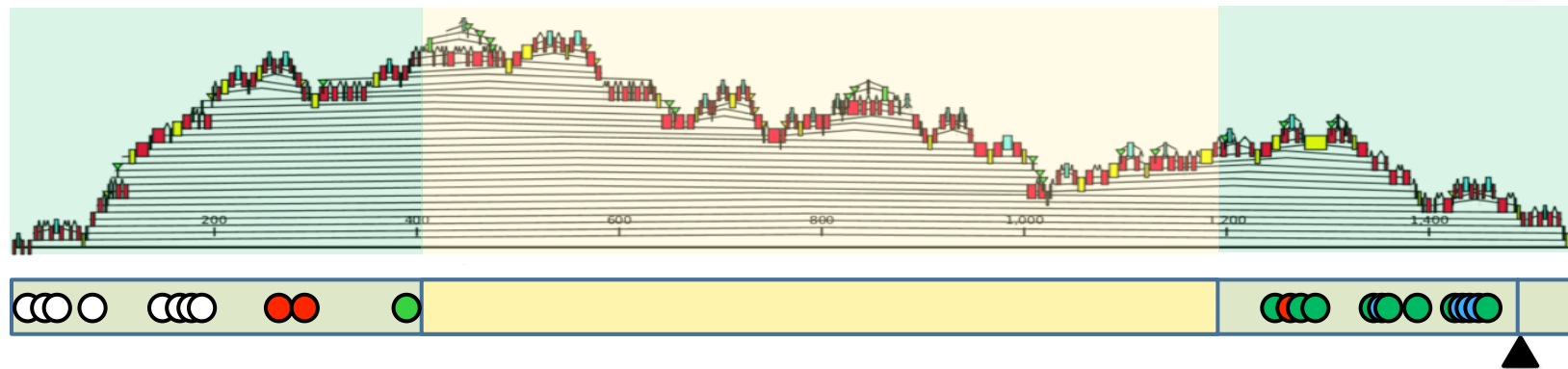

**D**  
pCH768

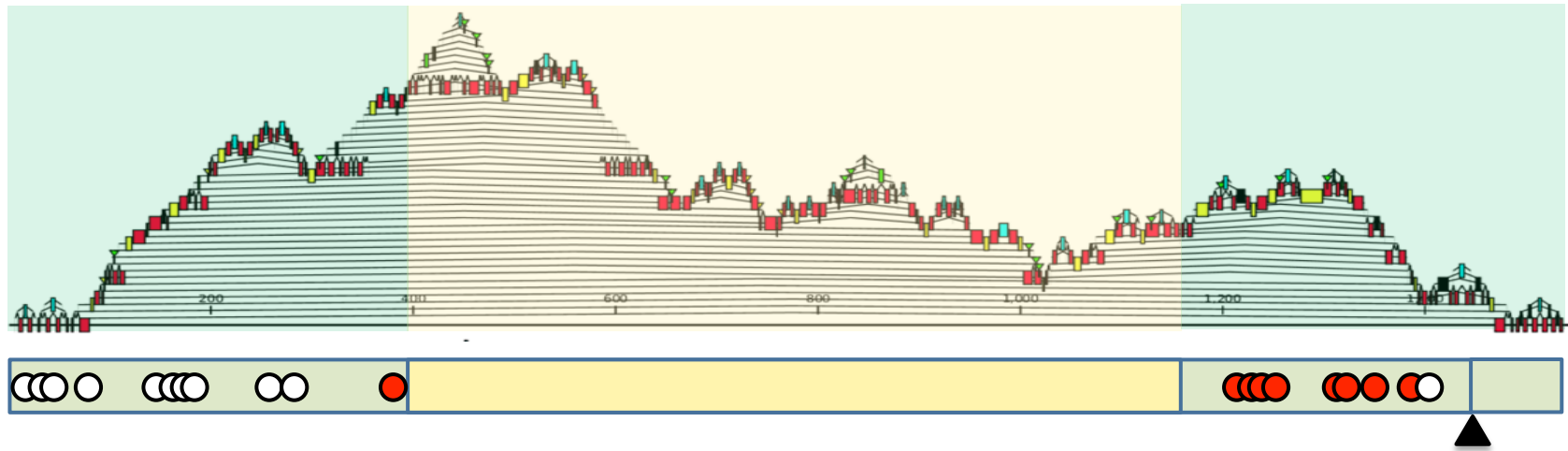

**E**  
pCH765

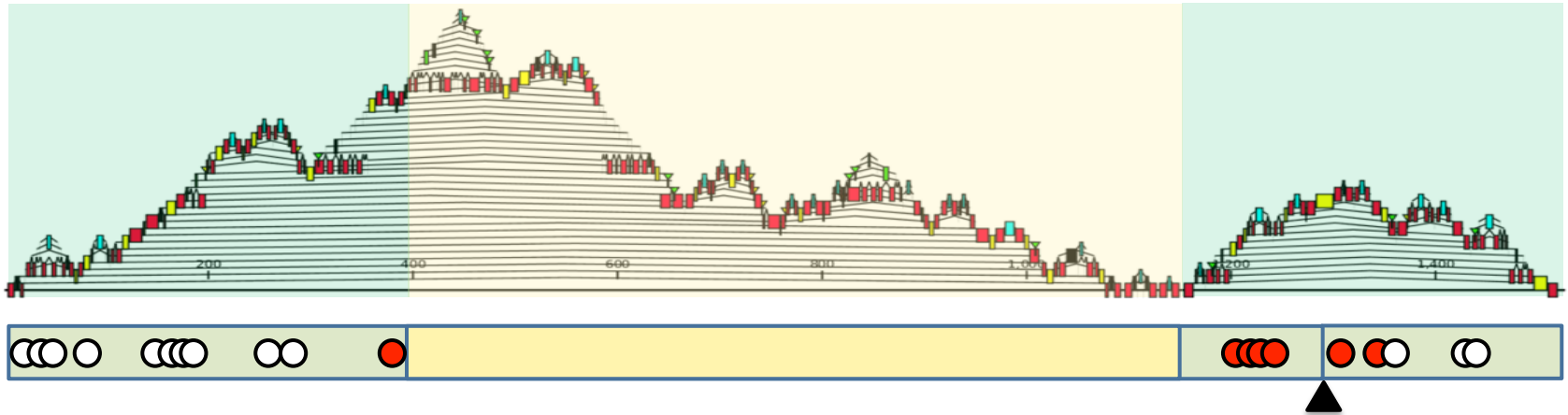

**F**  
pCH737

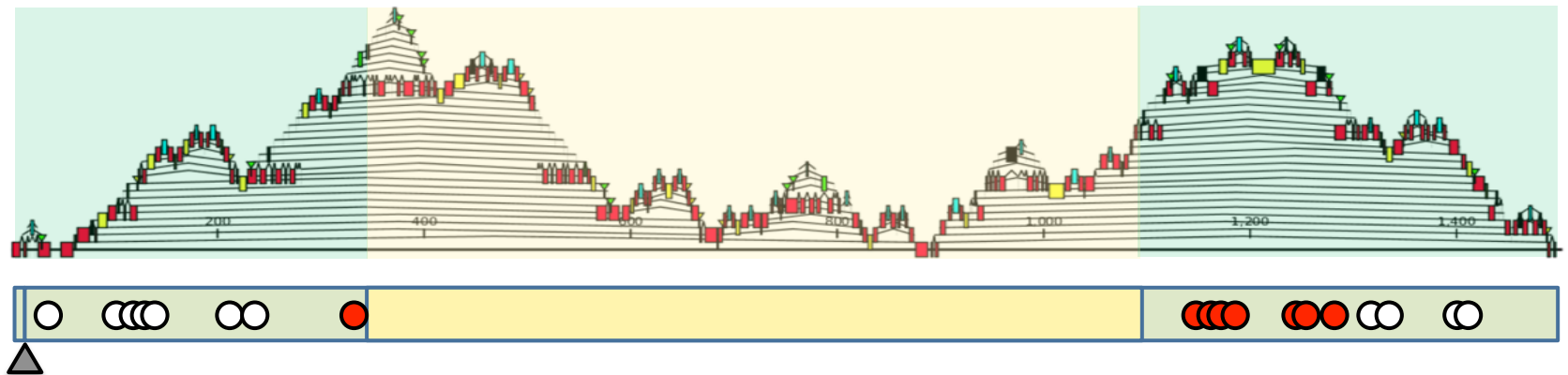

**G**

pCH754

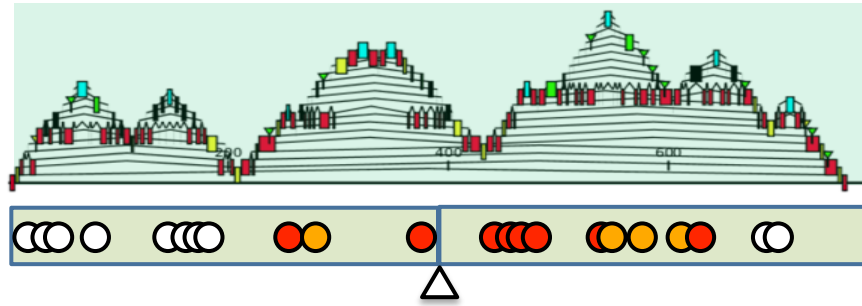**H**

pCH753

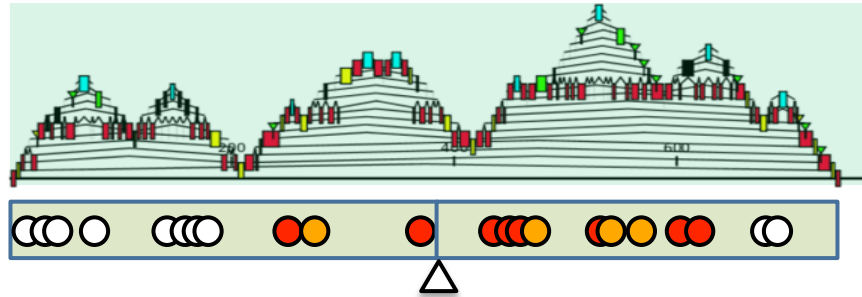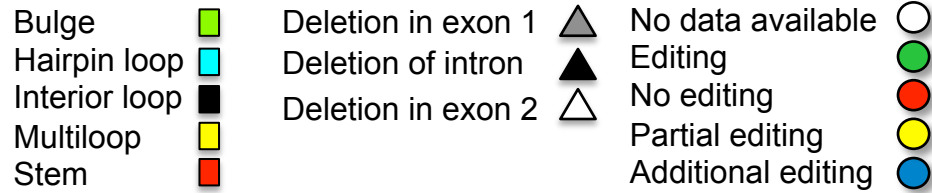

Supplement: Figure S4 — Models for RNA secondary structure. (A-H) Editing status of RNA from different plasmids and comparison with RNA secondary predictions. (PDF) [file pone.0082067.s004.pdf]
