# Supplementary material for: Deletions in cox2 mRNA Result in Loss of Splicing and RNA Editing and Gain of Novel RNA Editing Sites
Source: PLoS One. 2013 Dec 4;8(12):e82067. doi: 10.1371/journal.pone.0082067 (PMC3852756; doi:10.1371/journal.pone.0082067)
Supplement: File S1 — This file contains all supplementary tables. (DOCX) [file pone.0082067.s005.docx]

**Table S1: Oligonucleotides used**

| **Name** | **Sequence (5‘ → 3‘)** | **Specificity (Position)** |
| --- | --- | --- |
| **CH2279** | GGACATCAATGGTAT**T**GGAGTGCGCCTCTTAACG | *Z. mays, cox2,* Exon 1 (+370) |
| **CH2280** | CGTTAAGAGGCGCACTCC**A**ATACCATTGATGTCC | *Z. mays, cox2,* Exon 1 (+403) |
| **CH2294** | GCAACACCTATGATGCAAGGAATCATTGACTTAC | *Z. mays, cox2,* Exon 1 (+88) |
| **CH2319** | GCTATACCATCGTTTGCTCTGTTATAC | *Z. mays, cox2,* Exon 1 (+295) |
| **CH2320** | TTCACGACATCCCTTGCTTCTC | *Z. mays, cox2,* Intron (+964) |
| **CH2321** | TGGCTGGTACAACCACTCTATTGTC | *Z. mays, cox2,* Intron (+444) |
| **CH2322** | AAGCATCTGGCTTACCGGTCATC | *Z. mays, cox2,* Exon 2 (+1317) |
| **CH2328** | ACGAAGAATCATCCCATCTTTCAAAGCAAAGG | *Z. mays, cox2,* Exon 1 (+12) |
| **CH2337** | CCGATACCATTGATGTCCAATAGCTTTGATAG | *Z. mays, cox2,* Exon 1 (+387) |
| **CH2338** | CCTTATGAGTATTCGGACTATAACAGTTCCGATG | *Z. mays, cox2,* Exon 2 (+1182) |
| **CH2355** | CTTATGAGTATTCGGACTATAACAGTTCCGATGAACAG | *Z. mays, cox2,* Exon 2 (+1183) |
| **CH2356** | TCCGATACCATTGATGTCCAATAGCTTTGATAG | *Z. mays, cox2,* Exon 1 (+388) |
| **CH2358** | CTCCTCCTT**T**TTCGCTTCGGGGAC | *Z. mays, cox2,* Intron (+831) |
| **CH2359** | CGAAGCGAA**A**AAGGAGGAGCAGGAAC | *Z. mays, cox2,* Intron (+849) |
| **CH2381** | GAAATTATTCGGACCATTTTTCCTAGTGTCATTC | *Z. mays, cox2,* Exon 1 (+250) |
| **CH2382** | CATCCGTGATACGAAAACCAAAATCAGAATG | *Z. mays, cox2,* Exon 1 (+174) |
| **CH2383** | CTGTACCTTCCTCAGGTGTCAAATG | *Z. mays, cox2,* Exon 2 (+1369) |
| **CH2384** | AATGGTCCGAATAATTTCGATAGTAGTTC | *Z. mays, cox2,* Exon 1 (+264) |
| **CH2385** | AAATCTCACTGCACTGACCATAG | *Z. mays, cox2,* Exon 2 (+1476) |
| **CH2386** | GGGTATCCAATCAATTAATCCTCCAAAC | *Z. mays, cox2,* Exon 2 (+1543) |
| **CH2387** | TAGAAGCAGTGACTTTGAAAGATTATG | *Z. mays, cox2,* Exon 2 (+1510) |
| **FK357** | GATTAATTGATTGGATACCCGAGAAC | *A. thaliana, cox2,* Exon 2 (+2106) |
| **FK638** | GCGCTTTATGGCATTTCCACTATA | *A. thaliana, cox2,* Exon 1 (+176) |
| **FK789** | CCAATCCGCATAATCTTTCAAAGTC | *Z. mays, cox2,*Exon 2 (+1544) |
| **FK864** | GGGGTTAATTAAGCCTTACCACACCAACCACC | *A. thaliana cox2, 5´UTR* |
| **FK865** | GGGGATTTAAATATTAAATCTCACTGCACTGACCATAG | *Z. mays, cox2, exon* |
| **IH977** | CACCTTACTCTTAAATTTCCGCTATTTAAATATTA | *A. thaliana cox2,* 3’-NCS |
| **NB852** | CGCTTTATGGCATTTCAACGAGC | *Z. mays, cox2*, exon (+183) |

mutated nucleotides are given in bold letters

**Table S2: Vectors used in this study**

| **Plasmid** | **Description** | **Oligonucleotides used** |
| --- | --- | --- |
| **pCH736** | *Z. mays cox2*-ORF (Δ 99 bp exon 2, C385→T),  *A. thaliana* *cox2* 3‘-/5‘-NCS | CH2279/CH2280 |
| **pCH737** | *Z. mays cox2*-ORF (Δ 75 bp exon 1, +13),  *A. thaliana* *cox2* 3‘-/5‘-NCS | CH2294/CH2328 |
| **pCH753** | *Z. mays cox2*-ORF (Δ 1 bp-intron-13 bp, +388),  *A. thaliana* *cox2* 3‘-/5‘-NCS | CH2337/CH2338 |
| **pCH754** | *Z. mays cox2*-ORF (Δ intron, +389),  *A. thaliana* *cox2* 3‘-/5‘-NCS | CH2355/CH2356 |
| **pCH755** | *Z. mays cox2*-ORF (Δ 99 bp exon 2, +1477; C840→T),  *A. thaliana* *cox2* 3‘-/5‘-NCS | CH2358/CH2359 |
| **pCH756** | *Z. mays cox2*-ORF (Δ 99 bp exon 2, +1477; C385→T, C840→T), *A. thaliana* *cox2* 3‘-/5‘-NCS | CH2358/CH2359  CH2279/CH2280 |
| **pCH765** | *Z. mays cox2*-ORF (Δ 52 bp exon 2, +1317),  *A. thaliana* *cox2* 3‘-/5‘-NCS | CH2322/CH2383 |
| **pCH767** | *Z. mays cox2*-ORF (Δ 66 bp exon 2, +1477),  *A. thaliana* *cox2* 3‘-/5‘-NCS | CH2385/CH2386 |
| **pCH768** | *Z. mays cox2*-ORF (Δ 33 bp exon 2, + 1477),  *A. thaliana* *cox2* 3‘-/5‘-NCS | CH2385/CH2387 |
| **pHS571** | *Z. mays cox2*-ORF (Δ 99 bp exon 2, +1477),  *A. thaliana* *cox2* 3‘-/5‘-NCS | FK 864/865 |
| **pNB475** | *Z. mays cox2*-ORF,  *A. thaliana* *cox2* 3‘-/5’- NCS | ([Bolle and Kempken, 2006](#_ENREF_2)) |

**Table S3: Differences in RNA structural elements in different RNA sequences upon mutation**

Excel data file with details of differences among the secondary structure predictions shown in Figure S4.
